# Nagaland health assessment: High mortality rates and difficulty accessing essential health services in Lahe Township, Republic of the Union of Myanmar

**DOI:** 10.1371/journal.pone.0216925

**Published:** 2019-05-14

**Authors:** Win Le Shwe Sin Ei, Than Lwin Tun, Chit Htun, Etienne Gignoux, Kyaw Thu Swe, Andrea Incerti, Derek C. Johnson

**Affiliations:** 1 Medecins Sans Frontieres, Geneva Operational Center, Genève, Switzerland; 2 Sagaing Regional Health Department, Ministry of Health and Sports Myanmar, Yangon Myanmar; Makerere University School of Public Health, UGANDA

## Abstract

**Introduction:**

Lahe Township belongs to Myanmar`s Naga Self-administered Zone, which is one of the most remote and mountainous areas in Myanmar. However, the limited health data available for the region suggests that there could be neglected health needs that require attention. The purpose of this study was to assess the health status of the population of Lahe Township.

**Methods:**

A cross-sectional study design incorporating a two-stage cluster sampling methodology recommended by the WHO was used to conduct a household level survey. In the first stage, 30 village clusters were selected from all villages situated in the Lahe Township through systematic sampling with probability of selection proportional to the population size of each village based on the 2014 Myanmar census. In the second stage, a GPS-based sampling method was used to select 30 households within a village cluster. The head of the household completed the survey for all members of the household. Questionnaires inquired about maternal health, mortality, morbidities, childhood nutritional status, access to health care, and water & sanitation. The resulting data was stratified by urban/rural status.

**Results:**

Data was collected on 5,929 individuals living in 879 households, of which 993 individuals (16.7%) were children 5 years old or younger. The median age was 18.0 (IQR 8.0–35.0). Children 15 years old or younger represented 44.7% of the population. 19.8% of households reported at least 1 household member sick during the previous 30 days. The crude mortality rate per 10,000 people per day was 0.58 (95% CI: 0.48–0.69). The under 5 mortality per 10,000 people per day was 0.74 (95% CI: 0.50–1.06). Only 46.7% of households could access a hospital if there was a need.

**Conclusion:**

Our results demonstrate a high rate of mortality and the inability to access healthcare in Lahe Township, which should be addressed to prevent further deterioration of health.

## Introduction

Myanmar has one of the fastest growing economies in South East Asia, with a gross national income of $1,455 in 2017 and a gross domestic product (GDP) growth rate of 6.4% in 2016/2017(1). Poverty rates have decreased dramatically from 44.5% in 2004 to 26.1% in 2015[1] suggesting that Myanmar`s economy is rapidly expanding. However, despite Myanmar`s economic growth, Myanmar`s total expenditure on health care per capita is just USD $103 and total expenditure on health as a percentage of GDP is only 1.2%, significantly lower than the World Health Organization recommendation of 5% of GDP [2]. Myanmar`s inadequate health care funding has hindered the development of better health care relative to other Association of Southeast Asian Nations countries in the South East Asian region [3][4][5][6].

The health of Myanmar’s citizens is considerably worse for individuals living in Myanmar`s more remote regions [7]. Lahe Township, the area of focus for this study, belongs to the Naga Self-administered Zone in the Hkamti district of Sagaing Myanmar region, which is one of the most isolated, remote, and mountainous areas in Myanmar [8]. The Naga Self-administered Zone was established by the 2008 Myanmar constitution and consists of three townships; Lahe, Leshi, and Nanyun. Tribal administration in Lahe is normally led by a council of elders. However, the Myanmar government appoints village officials known *gaonburas* who are in charge of government administration, collecting taxes, and generally act as a go-between for tribes and the Myanmar government. Religion holds a central role in the Naga community, with the majority of individuals in Lahe Township identifying as Christian, in contrast to the majority of Myanmar citizens identifying as Buddhist [9]. Little is known about the health status of individuals living in Lahe Township. However, the information that is known suggests that Lahe Township is suffering from poor indicators of health. Lahe Township has 54,357 inhabitants[5], 10.3% of which live in urban settings. Only 20.6% of individuals older than 15 years are literate, which is significantly lower than the national literacy rate of 89.5% [6]. The majority of households (78%) in Lahe Township are bamboo huts, only 24.0% of households have access to improved toilet facilities, while 30.1% have no access to any type of toilet at all. Improved drinking water sources are accessible by 36.0% of all households in Lahe Township [5]. Life expectancy and mortality data are only available at district level. However, life expectancy at birth in Hkamti district is 62.0 years, which is the second lowest of all districts in Sagaing and is four years below the national-level estimate of 66.0 years [10]. Infant mortality was estimated as 77.8 per 1,000 live births, the number of deaths of children under 5 years of age was estimated as 90.3 deaths per 1000 live births, both among the two highest in all districts of Sagaing and both are notably higher compared to national level estimates [5].

This health assessment of Lahe township was conducted as a standalone project whose purpose was to help inform the future operational health activities of *Medecins Sans Frontiers* (MSF) in the region. The aim of this study was to assess the health status of the population of Lahe Township by measuring the following self-reported indicators: household mortality rates for the past one year, morbidity in the last 30 days, access to health care, maternal health, childhood nutritional status, and access to water & sanitation facilities.

## Methods

### Study area and population

Data was collected in Myanmar`s Lahe Township during the end of the dry season between February 13^th^ and March 21^st^ 2018. Access to the majority of villages in Lahe Township is only possible during the dry season and not consistently during the year due to the significant risk of dirt roads along mountain routes becoming inaccessible during heavy rains. According to Myanmar`s 2014 census [5], the population in Lahe Township consists of 54,357 people living in 8,932 households which are distributed among 97 villages. Using the national growth rate of 0.89% [5], the current study population is estimated at around 55,329 individuals.

Lahe Township`s health care delivery system is organized through hierarchical levels. Lahe Township has one township hospital, one station hospital, one mother and child health center, six rural health centers, and thirty sub-centers [2]. In the rural areas of Lahe Township, rural health centers and sub-centers are responsible for providing primary health care for the community and every facility is required to be staffed with at least one health care worker and one midwife. The sub-centers provide services directly to the village they are located in and to a network of 1–5 surrounding villages. Patients in need of secondary care are referred to one of the following health facilities: Station Hospital Unit, Township Hospital, District Hospital (Hkamti), or to a Specialist Hospital outside of the district[5].

### Sampling

A cross-sectional study design incorporating a two-stage cluster sampling methodology recommended by the WHO was used in this study [11]. In the first stage, 30 village clusters were selected from all villages listed in the Myanmar census within the Lahe Township area, where one village cluster was defined as a group of 30 household within a village. Cluster allocation to a village was conducted through systematic sampling with probability of selection proportional to the population size of each village based on the 2014 Myanmar census. Because the selection of village clusters was proportional to the size of a village, it was possible for larger villages to have multiple clusters assigned to it. In total, 30 village clusters, representing 28 villages, were selected for surveying. “Ground-truthing”, whereby a survey team was dispatched to a village selected for sampling, was used to ensure that a selected village existed.

In the second stage, a GPS-based sampling method was used to select 30 households within a village cluster. An electronic outline of each village selected was generated using QGIS software version 2.18 [12]. In each cluster selected for sampling, 30 GPS-points representing 30 households (1 village cluster) for surveying were randomly distributed within the boundaries of the village. Teams used GPS devices to find the respective random GPS point and surveyed the household that was identified to be physically closest to it. “Ground-truthing” methodology was used to ensure that houses which appear on the satellite imagery exist.

### Inclusion and exclusion criteria

The head of each selected household was asked to participate in the study if they lived in a randomly selected household and at least one household member was 18 years old or older and was able to answer our survey question. A household was excluded from the study if the study team was unable to locate the potential household head after two attempts to make contact or nobody 18 years old or older was able to answer the survey questions.

### Data collection

Two survey groups were recruited from Lahe Township to conduct interviews. Each group consisted of 3 data collection teams (one male and one female per team) and one group supervisor. Village leaders from each village were informed about the survey objectives before the survey began. In households selected for surveying, the purpose of the study was explained to the head of the household, in either Myanmar or Nagamese according to the preferences of the head of the household.

The study questionnaire included sections on household demographics, mortality, morbidity, access to health care, nutritional status, and water & sanitation. These sections were chosen to be included in the questionnaire in order to help MSF better target their primary health care activities in Lahe township. Questionnaires were first drafted in English, than translated into Myanmar, and then back translated into English to insure the quality of the translation. Survey questions were translated into Nagamese in the field for household members who preferred to speak the Nagamese language. Study staffs responsible for implementing the survey in the field were asked to review the questionnaire for any cases of poor translations. The head of the household participating in the survey answered questions for all individuals in the household. In order to reduce potential seasonal biases when recalling health outcomes, both the rainy and dry seasons of Lahe Township were included in survey unless noted otherwise. Because mortality is an easily remembered event, the recall period for a death in the household was 12 months (approximately 365 days). The recall period for an illness in the household covered 30 days prior to the survey because most illnesses are not as memorable as a death in the household. The total recall period for pregnancies was the previous two years. For the purpose of this study, individuals living in Lahe town, the capital of Lahe Towship, were considered to be living in an urban area and all other individuals were considered to be living in rural areas. Causes of mortality were self-reported and were ascertained using the following question “Now I am going to ask about the household members who have died in the last year. Please answer the following questions for each member of the household who has died. What was the cause of death?” Individuals were given the following options when responding to the potential causes of a death: 1) Watery diarrhoea 2) Bloody stool 3)Suspected malaria (fever and chills) 4) Respiratory tract infection 5) Malnutrition 6) Suspected measles (rash and fever) 7) Maternal death (death during pregnancy, childbirth, or within 6 weeks post-partum) 8) Newborn death (during childbirth or within 28 days post-partum 9) Injuries/accident/trauma/violence 10) Non-communicable chromic disease 11) Other 12) don’t know 13) refuse. Causes of morbidity were self-reported and were ascertained using the following question “Now I am going to ask about the illnesses and health problems that individuals in your household may have been experiencing. For each person in your house that is either currently sick or was sick in the last 30 days please answer the following question. What was the cause of the disease?” Individuals were given the following options when responding to the potential causes of a death: 1) Watery diarrhoea 2) Bloody stool 3)Suspected malaria (fever and chills) 4) Respiratory tract infection 5) Malnutrition 6) Suspected measles (rash and fever) 7) gyn-obatetrics 8) Injuries/accident/trauma/violence 9) Non-communicable chromic disease 10) Muskuloskeletal pain (general body pain, back and joint pain) 11) Skin disease 12) Other 13) don’t know 14) refuse. Individuals who displayed signs of severe illness were referred to Lahe Township hospital by our study staff for additional medical care. The mid-upper arm circumference (MUAC) was used to assess the nutritional status of children between the ages of 6–59 months [13].

### Data analysis

Data analysis was conducted using R 3.4.1 statistical software [14]. All indicators (proportions, ratio, rates) were stratified by urban/rural status. Where appropriate, differences in proportions were measured using Pearson χ2 test and differences in means and medians were measured using Students T-tests and Mann-Whitney U tests. Mortality rates were calculated as per 10,000 people per day, according to SPHERE international standards [15]. Mid-upper arm circumference scores were classified according to the following designations based on World Health Organization guidelines [16]: 1) greater than 135mm 2) greater than 125mm to 135mm 3) 115mm to 125mm 4) Less than 115mm. Severe Acute Malnutrition was defined as a MUAC score less than 115mm and Moderate Acute Malnutrition was defined as a MUAC score between 115mm to 125mm.

### Ethical approval

Ethical approval to conduct this study was given by the Medecins Sans Frontieres Ethics Review Board in December 2017 and by the Department of Medical Research, Myanmar Ministry of Health and Sports in January 2018. A mid-term report on project activities and copies of all signed informed consents were delivered to the Myanmar Ministry of Health, Department of Medical Research in order to demonstrate this study`s ethical compliance. Written consent was obtained before the interviews were conducted. If the respondent was illiterate, the consent form was read out loud and verbal consent was obtained. Consent was witnessed and the consent form was co-signed by the person conducting the interview.

## Results

In total, 879 households were surveyed, representing 5,929 individuals who were either living or have died during the previous year in these households. In total, 97.6% of households agreed to participate in the survey (879 / 900) (Table 1).

**Table 1:** Demographic characteristics of Lahe Township stratified by urban & rural status*^%^.

	Overall (%)	Urban Population (%)	Rural Population (%)	P-value
Number of households interviewed	879	89 (10.1)	790 (89.9)	n/a
Total number of people living in households interviewed	5,929	518 (8.7)	5411 (91.3)	n/a
Average number of people per household				
Mean (SD)	6.8 (2.9)	5.8 (1.9)	6.8 (2.9)	0.001
Median (IQR)	6.0 (5.0–8.0)	5.5 (4.0–7.0)	7.0 (5.0–9.0)	
Age				
Mean (SD)	23.5 (19.7)	20.7 (17.1)	23.8 (19.9)	0.001
Median (IQR)	18.0 (8.0–35.0)	17.0 (7.0–30.0)	18.0 (8.0–35.0)	
Sex				
Male	3006 (50.9)	255 (49.2)	2751 (51.1)	0.42
Female	2898 (49.1)	263 (50.8)	2635 (48.9)	
Male / Female sex ratio	1.04	0.96	1.04	
Number of children <5yrs	993 (16.7)	98 (18.9)	895 (16.6)	0.17
Number of children <15yrs	2641 (44.6)	243 (46.9)	2398 (44.4)	0.26
Number of children <5yrs per household				
Mean (SD)	1.2 (1.1)	1.1 (0.9)	1.2 (1.0)	0.48
Median (IQR)	1.0 (0–2.0)	1.0 (0–2.0)	1.0 (0–2.0)	
Ethnicity of head of household				
Naga	25 (2.9)	1 (1.1)	24 (3.1)	0.001
Lai Naung	305 (34.7)	31 (34.8)	274 (34.9)	
Ponny Nyan	162 (18.6)	11 (12.4)	151 (19.3)	
Gongvongpounyin	181 (20.7)	12 (13.5)	169 (21.6)	
Khamnyiugan	124 (14.2)	3 (3.4)	121 (15.4)	
Tang Shang	38 (4.4)	13 (14.6)	25 (3.2)	
Other	38 (4.4)	18 (20.2)	20 (2.6)	
Head of household education level				0.001
No education	608 (69.3)	36 (40.5)	572 (72.6)	
Primary	167 (19.1)	16 (17.9)	151 (19.2)	
Secondary	89 (10.2)	32 (35.9)	57 (7.2)	
College/University	13 (1.5)	5 (5.6)	8 (1.0)	

*Lahe Township was considered “Urban” during data analysis.

^%^The urban/rural variable was based on village cluster, which is the same factor for clustering

### Demographics

**Fig 1:**
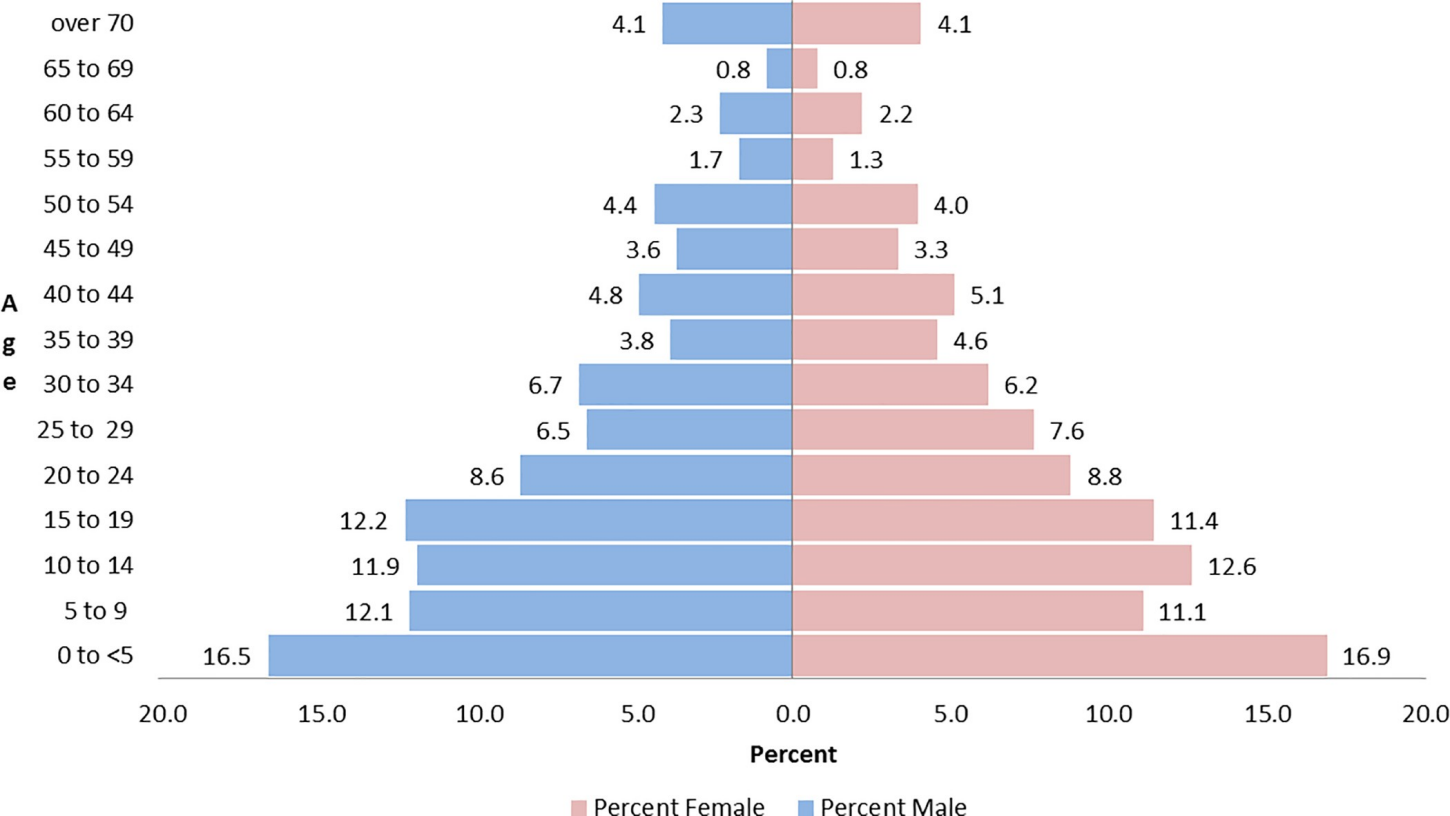
Lahe township population pyramid stratified by sex.

Of the 5,929 individuals represented in this survey, 993 individuals (16.7%) were children 5 years old or younger. The median number of people living in each household was 6.0 (IQR 5.0–8.0) (Table 1). The citizens of Lahe Township are predominantly young, with children 15 years old or younger representing 44.7% of the population (Fig 1). The median age of Lahe Township was 18.0 (IQR 8.0–35.0). Three ethnic groups represented 74.2% of the surveyed households: Lai Naung (34.9%), Gongvongpounyin (20.7%), and Ponny Nyan (18.6%) (Table 1). Over two thirds of heads of households reported no formal level of education (69.3%), while 19.1% of household heads reported having some or completed a primary level of education (Table 1).

There were significant demographic differences between urban and rural households. The median household member`s age was significantly older in rural areas than in urban areas (Median age in urban vs. rural areas: 17.0 vs. 18.0, p-value 0.001) (Table 1). Heads of households from urban areas were over four times more likely to have a secondary level of education or higher compared to heads of households from rural areas (Proportion of heads of households with secondary level of education or higher in urban vs. rural: 35.9 vs. 7.2, p-value 0.001). Individuals from rural areas were more likely to report belonging to the Lai Naung (34.9%), Ponny Nyan (19.3%), and Gongvongpounyin (21.6%) ethnicities, while individuals from urban areas were most likely to report their ethnicity as Lai Naung (34.7%), Tang Shang (14.6), and “Other” (20.2) (p-value 0.001) (Table 1).

### Mortality

Of the 5,292 individuals represented in this survey, 112 (2.1%) individuals were reported to have died within the previous year, of which 27 deaths were children under 5 years of age (2.7% of children under). The overall crude mortality rate (CMR) per 10,000 people per day was 0.58 (95% CI: 0.48–0.69 (Table 2). The largest causes of mortality reported by the head of household mortality were respiratory tract infections (RTIs) (34.1%), Watery diarrhea (8.2%), Non-communicable chronic diseases (8.2%), and “Other” (28.2%) (Table 3).

**Table 2:** Retrospective mortality rates in Lahe Township stratified by urban/rural status.

	Overall number of deaths	Overall death rate (95% CI)*	Number of deaths in urban areas	Death rate in urban areas (95% CI)*	Number of deaths in rural areas	Death rate in rural areas (95% CI)*	P-value
Crude mortality	112	0.58 (0.48–0.69)	6 (5.4)	0.32 (0.13–0.66)	106 (94.6)	0.53 (0.44–0.64)	0.20
Under 5 mortality	27	0.74 (0.50–1.06)	0	0 (0)	27 (100.0)	0.82 (0.55–1.18)	0.25
Sex specific mortality**							0.09
Male	55	0.50 (0.38–0.64)	5 (83.3)	0.53 (0.19–1.18)	50 (54.4)	0.54 (0.41–0.71)	
Female	43	0.41 (0.29–0.54)	1 (16.7)	0.10 (0.01–0.51)	42 (45.7)	0.43 (0.31–0.58)	
Age**							0.02
0 to 14	37	0.42 (0.30–0.57)	0 (0)	0 (0)	37 (40.2)	0.46 (0.33–0.63)	
15 to 24	12	0.14 (0.07–0.23)	2 (33.3)	0.56 (0.09–1.8)	10 (10.9)	0.25 (0.12–0.43)	
25 to 34	7	0.24 (0.11–0.47)	2 (33.3)	0.58 (0.1–1.8)	5 (5.4)	0.19 (0.08–0.43)	
35 to 44	11	0.56 (0.29–0.96)	1 (16.7)	0.53 (0.03–2.5)	10 (10.9)	0.56 (0.28–0.98)	
45 to 54	11	0.66 (0.35–1.14)	0 (0)	0 (0)	11 (11.9)	0.71 (0.37–1.22)	
55 to 64	6	0.74 (0.30–1.53)	0 (0)	0 (0)	6 (6.5)	0.77 (0.31–1.58)	
65+	14	1.3 (0.76–2.13)	1 (16.7)	2.11 (0.11–8.9)	13 (14.1)	1.29 (0.72–2.12)	

*Deaths per 10,000 people per day

**112 deaths were reported. However on 98 deaths had additional information for age and sex

**Table 3:** Cause of death in Lahe Township by age group*.

**Cause of death**	**Overall**	**Under 5yr**	**5yrs or older**
Watery diarrhea	7 (8.2)	1 (4.2)	6 (9.8)
Bloody stool	4 (4.7)	0 (0)	4 (6.6
Suspected malaria	7 (8.2)	1 (4.2)	6 (9.8)
Respiratory tract infection	29 (34.1)	12 (50.0)	17 (27.9)
Suspected measles	3 (3.5)	3 (12.5)	0 (0)
Injuries / Accident/Trauma/Violence	4 (4.7)	2 (8.3)	2 (3.3)
Non-communicable chronic diseases	7 (8.2)	0 (0)	7 (8.2)
Other	24(28.2)	5 (20.8)	19 (31.2)

* There was no cause of death listed for 3 children under 5 and 24 individuals older than 5

Age was significantly associated with mortality when stratified by urban/rural status. Within the rural stratification, half of mortality happened among individuals younger than 25 years old (51.1%). Among households living in urban areas the majority of deaths occurred in individuals between the ages of 15 to 34 (66.6%) (p-value 0.02) (Table 2). There was no significant differences in the causes of mortality when stratified by urban/rural status.

### Morbidities

One hundred and ninety one people (3.3% of all individuals) from 176 households (20.1% of all households) were reported as being sick at least once during the last 30 days (Table 3). The most commonly reported diseases were: Respiratory tract infection (RTI) (29.7%), Musculoskeletal pain (22.1%), Watery diarrhea (12.2%), and “Other” (24.4%) (Table 4). Of the individuals who reported an RTI in the past 30 days, 33 (64.7%) reported their respiratory infection lasting 2 weeks or longer. Of the individuals who had respiratory illnesses lasting 2 weeks or longer, 13 (34.6%) reported having a fever, 4 (15.4%) reported coughing up blood, 5 (23.1%) reported unexplained weight loss, 6 (26.9%) reported night sweats (Table 4). Only 57% of individuals reporting a cough lasting 2 weeks or longer took medication for the illness, of which 28.6% took the medication for 6 months or longer.

**Table 4:** Morbidity indicators in Lahe Township stratified by urban & rural status.

	Overall (%)	Urban Population (%)	Rural Population (%)	P-value
Number of individuals in a household sick during the last 30 days				0.21
0	704 (79.9)	76 (85.4)	627 (79.4)	
1	164 (18.7)	12 (13.5)	152 (19.2)	
2	9 (1.1)	0 (0)	9 (1.1)	
3+	3 (0.3)	1 (1.2)	2 (0.3)	
Type of illness				0.92
Watery diarrhea (3 or more loose stools in 24 hours)	21 (12.2)	2 (10.5)	19 (11.3)	
Bloody stool	3 (1.7)	0 (0)	3 (1.8)	
Suspected malaria (fever and chills)	6 (3.5)	0 (0)	6 (3.6)	
Respiratory tract infection	51 (29.7)	8 (42.1)	43 (25.6)	
Gyn-obstetrics	1 (0.6)	0 (0)	1 (0.6)	
Injuries / Accident/Trauma/Violence	3 (1.7)	0 (0)	3 (1.8)	
Non-communicable chronic diseases	4 (2.3)	0 (0)	4 (2.4)	
Muskuloskeletal pain	38 (22.1)	3 (15.8)	35 (20.8)	
Skin diseases	3 (1.7)	0 (0)	3 (1.89)	
Other (Specify)	42 (24.4)	5 (26.3)	37 (22.0)	
If you have an RTI, how long have you been coughing				0.25
Less than 2 weeks	5 (13.2)	0 (0)	5 (16.1)	
2 weeks or longer	33 (86.8)	7 (100.0)	26 (83.9)	
If you have an RTI, do you have any of the following symptoms				0.22
Fever	13 (46.4)	6 (75.0)	7 (35.0)	
Coughing up blood	4 (14.3)	1 (12.5)	3 (15.0)	
Unexplained weight loss	5 (17.9)	0 (0)	5 (25.0)	
Night sweats	6 (21.4)	1 (12.5)	5 (25.0)	

### Access to health care

When asked if a household member who was ill visited a health care provider for their illness during the last 30 days, 42.4% responded yes (Table 5). Over half of the households surveyed said they would not be able to access a hospital if they needed to (53.6%). When a household with an individuals who was sick during the last 30 days was asked “Where did he/she seek health care for their illness during the last 30 days”, approximately three quarters of households that were able to access health care reported first going to either a rural health center /sub-center (43.3%) or a maternal and child health center (30.4%) when someone is sick in their household (Table 5). For individuals who were unable to access health care when a household member was sick during the last 30 days, transportation and transport related costs were reported as the main reasons why an individual could not access health care when sick (40.3%). Lack of staff at a health center was also a common reason why an individual did not access health care (14.0%). Medical care was sought prior to death in 47.6% of cases (Table 5). Transportation and transportation related costs were the biggest reasons why an individual didn’t access health care prior to death (34.5%) (Table 5).

**Table 5:** Access to care indicators in Lahe Township stratified by urban & rural status.

	Overall (%)	Urban Population (%)	Rural Population (%)	P-value
Ability to go to a hospital if there is a need				0.001
Yes	410 (46.7)	72 (80.9)	338 (42.8)	
No	469 (53.3)	17 (19.1)	452 (57.2)	
Why can you not go to a hospital if there is a need				0.03
No transport	52 (48)	0 (0)	52 (52.0)	
Transport is too expensive	28 (26.2)	7 (100.0)	21 (21.1)	
Doctor costs are too expensive	2 (1.9)	0 (0)	2 (2.0)	
Lack of trust in health providers	3 (2.8)	0 (0)	3 (3.0)	
Service/staff is not available at the clinic	2 (1.9)	0 (0)	2 (2.0)	
Staff is rude/rejecting/discriminating	14 (13.1)	0 (0)	14 (14.0)	
Other	4 (3.7)	0 (0)	4 (4.0)	
Did a household member who was ill in the last 30 days seek medical care				0.19
Yes	53 (42.4)	1 (16,7)	52 (43.7)	
No	72 (57.6)	5 (83.3)	67 (56.3)	
If no, why did the household member who was ill in the last 30 days not seek medical care				0.91
Could not afford provider costs	7 (12.3)	0 (0)	7 (12.7)	
No transport	8 (14.0)	0 (0)	8 (14.6)	
Could not afford transport cost	8 (14.0)	0 (0)	8 (14.6)	
Long waiting list/time	1 (1.8)	0 (0)	1 (1.8)	
Do not trust in quality of service	3 (5.3)	0 (0)	3 (5.5)	
Service/staff not available	8 (14.0)	1 (50.0)	7 (12.7)	
Did not think it is important	2 (3.5)	0 (0)	2 (3.6)	
Did not know where to go	3 (5.3)	0 (0)	3 (5.5)	
Other	17 (29.8)	1 (50.0)	16 (29.1)	
Where do you go when sick				0.001
Local medicine shop	35 (4.2)	1 (1.1)	34 (4.5)	
Rural health center / Sub-center	364 (43.3)	0 (0)	364 (48.3)	
Auxiliary midwife	55 (6.5)	1 (1.1)	54 (7.2)	
Maternal child health center	256 (30.4)	79 (89.8)	177 (23.5)	
Lahe Township hospital / Myanmar Military hospital	30 (3.6)	0 (0)	30 (4.0)	
Private clinic	31 (3.7)	0 (0)	31 (4.1)	
Nowhere to go	56 (6.7)	5 (5.7)	51 (6.8)	
Other	14 (1.7)	2 (2.3)	12 (1.6)	
Was medical advice sought before an household member`s death^ç^				0.16
Yes	39 (47.6)	2 (100.0)	37 (46.3)	
No	43 (52.4)	0 (0)	43 (53.8)	
If yes, where was medical advice sought before an household member`s death				0.81
Local shop (self-medicate)	6 (9.0)	0 (0)	6 (9.4)	
Rural Health Centre, Sub-Centre	12 (17.9)	0 (0)	12 (18.6)	
Auxiliary midwife	1 (1.5)	0 (0)	1 (1.6)	
Traditional healer	2 (3.0)	0 (0)	2 (3.1)	
Maternal Child Health Centre	16 (23.9)	0 (0)	14 (21.9)	
Lahe Township Hospital	1 (1.5)	2 (66.7)	1 (1.6)	
Myanmar military hospital	4 (5.9)	0 (0)	4 (6.3)	
Other	25 (37.3)	1 (33.3)	24 (37.5)	
If no, why was medical advice not sought before an household member`s death				0.98
Could not afford provider costs	7 (11.3)	0 (0)	7 (11.5)	
No or difficult transport	5 (8.1)	0 (0)	5 (8.2)	
Could not afford transport cost	9 (14.5)	0 (0)	9 (14.8)	
Long waiting list/time	3 (4.8)	0 (0)	3 (4.9)	
Do not trust in quality of service	5 (8.1)	0 (0)	5 (8.2)	
Did not know where to go	3 (4.8)	0 (0)	3 (4.9)	
No time	6 (9.7)	0 (0)	6 (9.8)	
Not sick enough	2 (3.2)	0 (0)	2 (3.3)	
Other	22 (35.5)	1 (100.0)	21 (34.4)	

^ç^112 deaths were reported. However on 82 deaths had additional information on if medical advice was sought before death.

Individuals in urban areas were significantly more likely to be able to access a hospital if there was a need compared to rural individuals (80.9% vs. 42.8%). Over half of rural individuals said lack of transportation was the biggest reason they were unable to access a hospital if there was a need (52.0%). Only 53.8% of rural individual were able to access medical care before death (Table 5).

### Maternal health

Of the 2,898 women represented in this survey, 1364 (47.1%) were between the reproductive ages of 15 and 49 years old (Table 6). The number of households with at least one women of reproductive age currently pregnant at the time of the survey was 72 (10.2%). The number of households where at least one women of reproductive age gave birth within the last 2 years was 265 out of 731 households (148 households were missing information on pregnancy) (36.5%). The median age at first pregnancy was 20.0 years (IQR: 19.0–25.0) (Table 6). The majority (89.0%) of women reported not having a main source of contraception before they were pregnant. Less than half of women who gave birth in the last two years had any antenatal care visits (44.1%). Most women delivered their child at a home (85.2%) (Table 6).

**Table 6:** Maternal health indicators in Lahe Township stratified by urban & rural status.

	Overall (%)	Urban Population (%)	Rural Population (%)	P-value
Number of women aged 15–49				0.34
Yes	1364 (47.1)	134 (52.3)	1230 (49.2)	
Number of women currently pregnant per household				0.21
0	631 (89.8)	79 (95.2)	552 (89.0)	
1	71 (10.1)	4 (4.8)	67 (10.8)	
2	1 (0.1)	0 (0)	1 (0.2)	
Average age during first pregnancy				0.39
Mean (SD)	21.3 (4.9)	22.3 (5.2)	21.1 (4.9)	
Median (IQR)	20.0 (19.0–25.0)	20.0 (19.0–26.0)	20.0 (19.0–24.0)	
How many women have given birth in the last 2 years per household^$^				
0	466 (63.8)	48 (60.8)	418 (64.1)	
1	243 (33.2)	27 (34.2)	216 (33.1)	
2	19 (2.6)	4 (5.1)	15 (2.3)	
3+	3 (0.42)	0 (0)	3 (0.5)	
What was the main form of contraception before the household member became pregnant*^&^				0.02
No contraception	187 (89.0)	18 (78.3)	169 (90.4)	
Condom	2 (0.9)	1 (4.4)	1 (0.5)	
Injections	9 (4.3)	3 (13.0)	6 (3.2)	
Pill	9 (4.3)	1 (4.4)	8 (4.3)	
Other	3 (1.4)	0 (0)	3 (1.6)	
How many antenatal visits were made during the pregnancy*^+^				0.76
0	104 (55.9)	13 (48.2)	91 (57.2)	
1	34 (18.3)	4 (14.8)	30 (18.9)	
2	30 (16.1)	2 (7.4)	28 (17.6)	
3	14 (7.5)	8 (29.6)	6 (3.8)	
4+	4 (2.2)	0 (0)	4 (2.5)	
Where was the baby delivered				0.001
At home	213 (85.2)	16 (51.6)	197 (89.9)	
Rural Health Center / Sub-Center	4 (1.6)	0 (0)	4 (1.8)	
Lahe Township Hospital	12 (4.8)	9 (29.0)	3 (1.4)	
Other	21 (8.4)	6 (19.4)	15 (6.9)	

*For women who were pregnant within the last 2 years

^&^Data was missing for 55 of the 265 households with at least one woman who gave birth in the last 2 years(20.8%)

^+^Data was missing for 79 of 265 households with at least one woman who gave birth in the last 2 years (29.8%)

^$^Data was missing for 148 of 879 households surveyed (16.8%)

Women from urban areas were significantly more likely to have used contraception than women from rural areas (21.7% vs. 9.6%, p-value 0.02). The majority of women from rural areas delivered their baby at home, while only about half of women from urban areas delivered their baby at home (89.9% vs. 51.6%, p-value 0.001). Almost a third of women from urban areas delivered their baby at Lahe Township hospital (29.0%).

### Nutritional status

Of the 925 children between the ages of 6 to 59 months, 653 of them had a MUAC assessment for malnutrition (70.6%). The median MUAC score for children 6–59 months old was 145.0 (IQR: 138.0–153.0). Of the 653 children with a MUAC score, 2 (0.3%) had scores <115mm, 10 (1.5%) had scores between 115mm and 125mm, 113 (17.3%) children had scores between 125mm and 135mm, and 528 (80.9%) children has MUAC scores greater than 135mm (Table 7). There were no cases of oedem found during this study. While the difference in nutritional status by urban/rural status was not found to be significant, all cases of Severe Acute Malnutrition and Moderate Acute Malnutrition were found in rural areas.

**Table 7:** MUAC Scores for children 6–59 months in Lahe Township stratified by urban/rural status.

	Overall (%)	Urban Population (%)	Rural Population (%)	P-value
MUAC Score				0.68
>135mm	528 (80.9%)	50 (84.8)	478 (80.5)	
125mm– 135mm	113 (17.3%)	9 (15.2)	104 (17.5)	
115mm– 125mm	10 (1.5%)	0 (0)	10 (1.7)	
<115mm	2 (0.3%)	0 (0)	2 (0.3)	

### Water and sanitation

There were significant differences in the main source of water for households during the rainy and dry seasons. The most common sources of water during the rainy season were lakes/ponds (62.3%), followed by water from a tap/pipe (22.5%), and rivers/streams (8.3%) (Table 8). During the dry season, the most common sources of water were lakes/ponds (56.4%), followed by water from a tap/pipe (22.3%), and rivers/streams (12.6%) (Table 6). Boiling was the most popular way to treat water (79.1%). Most households could access a water source within 5 minutes (58.8%), followed by being able to access water within 5 to 30 minutes (26.3%), and being able to access a water source in over 30 minutes (2.7%) (Table 6). Several households had no access to a main source of water irrespective of season (12.2%). The majority of households used a latrine (improved & unimproved) as their main type of toilet facility (77.2%), followed by using no facility/bush/field (20.5%) (Table 8). Most latrines were shared between households, with 22.0% of households sharing a latrine with 1 to 5 other houses and 35.7% of households sharing a latrine with more than 5 houses. Soap was rarely used to wash hands after using a latrine (14.6%) and a large proportion of people reported never washing their hands after a toilet (39.2%) (Table 8).

**Table 8:** Water and sanitation in Lahe Township stratified by urban & rural status.

	Overall (%)	Urban Population (%)	Rural Population (%)	P-value
Main source of water during **Rainy** season				0.001
Tap/Pipped water	182 (21.8)	42 (47.2)	140 (18.8)	
Lake / Pond	504 (60.4)	31 (34.8)	473 (63.5)	
River / Stream	67 (8.0)	5 (5.6)	62 (8.3)	
Well/Spring	37 (4.4)	1 (1.1)	36 (4.8)	
Other	44 (5.3)	10 (11.2)	34 (4.6)	
Main source of water during **Dry** season				0.001
Tap/Pipped water	194 (22.2)	44 (50.0)	150 (19.2)	
Lake / Pond	491 (56.2)	28 (31.8)	463 (59.2)	
River / Stream	110 (12.6)	6 (6.8)	104 (13.3)	
Well/Spring	47 (5.4)	2 (2.3)	45 (5.8)	
Other	28 (3.3)	8 (9.1)	20 (2.6)	
Time it takes to access water source and come back				0.03
No regular access to water	107 (12.2)	8 (9.0)	99 (12.5)	
0 to 5min	517 (58.8)	58 (65.2)	459 (58.1)	
5 to 30min	231 (26.3)	19 (21.4)	212 (26.8)	
Over 30min	24 (2.7)	4 (4.5)	20 (2.5)	
How is the household water treated before drinking				0.001
Boiling the water	679 (79.1)	86 (96.6)	593 (77.1)	
Nothing / Drink directly	161 (18.8)	1 (1.1)	160 (20.8)	
Other	18 (2.1)	2 (2.3)	16 (2.0)	
Main type of toilet facility used by the household				0.001
Improved pit latrine	321 (36.5)	82 (92.1)	239 (30.3)	
Unimproved pit latrine	350 (39.8)	3 (3.4)	347 (43.9)	
No facility / Bush / Field	188 (21.4)	4 (4.5)	184 (23.3)	
Other	20 (2.3)	0 (0)	20 (2.5)	
How are hands washed after using the toilet				0.001
Water only	384 (43.9)	31 (34.8)	353 (44.9)	
Soap and water	123 (14.1)	43 (48.3)	80 (10.2)	
Do not wash hands	368 (42.1)	15 (16.9)	353 (44.9)	

More households in rural areas reported not having regular access to a water supply than urban populations (12.5% vs. 9.0%, p-value 0.03) (Table 8). During the dry season more urban individuals reported pipped water as their main source of water than rural individual (50.0% vs. 19.2%, p-value 0.001). This difference also extended into the rainy season (47.2% vs. 18.8%, p-value 0.001). Almost all urban individuals reported treating their water by boiling it (96.6%), while 20.8% of rural individuals reported not treating their water at all before drinking it. The majority of urban individuals used an improved pit latrine as a toilet (92.1), while rural individuals were likely to use an unimproved pit latrine (30.3%) or the bush/field (23.3%) as a toilet facility. Rural individuals were significantly more likely to not wash their hands (with or without soap) after using the toilet then urban individuals (44.9% vs. 16.9%, p-value 0.001) (Table 8).

## Discussion

### Demographics

Most of our sample`s demographic characteristics were similar to the demographics reported in the 2014 Myanmar census for the Sagaing Region [5]. However, our study`s findings on education deviated from the 2014 Myanmar census. Our study found that 69.3% of the heads of household surveyed have never received a formal education. However, when stratified by age to match the 2014 Myanmar census statistics on education. We found that 71.2% of our study`s heads of household who were 25 years old or older had never received a formal education. This is a lower percentage of individuals having never received a formal education than reported in the 2014 Myanmar census which found that 86.3% of individuals 25 years or older have never received a formal education. It is possible that the difference in education level between our study and the 2014 Myanmar census could be explained by the difference in urban/rural status of our study participants. When education was stratified by urban / rural status, the percentage of urban individuals who never received a formal education was similar between our study and the 2014 Myanmar census (40.4% vs. 41.2%)[8].

Children 15 years old or younger represented 44.7% of the population in Lahe Township, which could be a possible indicator of poverty in relation to Myanmar as a whole, as high birth rates and younger populations have been associated with poverty in other countries [17][18][8]. The percentage of children under 15 in our study is considerably higher than the percent of children under 15 in the Sagaing region (28.7%) and the national average (26.1%) [5]. Several studies have shown that high fertility rates are associated with a lack of wealth [19]. The Indonesian Family Life Survey found that each birth per woman reduced the likelihood of that female from participating in the labor force by 20 percent. This in turn reduced a households overall income, which in turn reduced the amount of money a family could spend on goods and services [20]. Data from Matlab, Bangladesh found that villages with the lowest birth rates had significantly higher earnings then villages with higher birth rates [21]. Lahe`s large percentage of children under 15 could suggest that Lahe Township is not only more impoverished, but that it is also developing at a slower pace than Myanmar overall.

### Morbidities

Approximately 1/5 of households reported at least one household member being sick in the last 30 days, with respiratory illnesses being the most common cause of morbidity. The large percentage of morbidity being caused by RTIs could be because this study took place during the winter months of the dry season, when the dry, cold weather was likely to increase exposure to indoor air pollution [22]. It is customary for houses in the Lahe Township to use indoor fire pits for cooking meals [23] and constant exposure to wood smoke could be a reason why RTIs are the largest source of morbidity and mortality in children under 5 years of age. Indoor air pollution caused by the burning of cooking fuels is well known to be associated with acute respiratory illness, such as pneumonia or bronchiolitis, in children under 5 years old [24][25]. In developing countries in particular, chronic exposure to indoor air pollution from open fires has been associated with 1.5 to 2 times the odds of developing an acute lower respiratory infection per year in children under 5 years of age [26]. Children are believed to be more prone to respiratory infections exacerbated by indoor air pollution for several reasons; the physiology of their lungs is less developed than adults lungs [27][28], children have a harder time fighting infection than adults because their immune systems are less developed, and children spend extended periods of time near fires during periods of cooking and heating. Given that most households in Lahe Township use open fires for cooking and heating, it is possible that indoor air pollution is a contributing factor to the number of RTIs reported in this study.

### Mortality

The crude mortality rate for the total the recall period was 0.58 (95% CI: 0.48–0.69) per 10,000 people per day, which is more than the emergency threshold set by SPHERE international standards for “Developing countries” (0.4/10,000/day) [15], but below the emergency threshold for “Least developed countries” (0.7/10,000/day). The mortality rate for children under 5 per 10,000 people per day was 0.74 (95% CI: 0.50–1.06), which is less than the emergency threshold for children under 5 years of age set by SPHERE international standards for “Developing countries” (0.9/10,000/day). It is also under the emergency threshold for “Least developed countries” (1.7/10,000/days) [15].

The primary cause of overall death was RTIs (34.1%) and RTIs represented 50.0% of deaths for children under 5 years old. RTIs are a common cause of death in developing countries [27][26]. Data from the Bangladesh Demographic and Health Survey from 2004 to 2014 found that consistent exposure to household pollution from cooking fuels increased the risk of neonatal mortality by 46% [29] and data from the 2013 Pakistan Demographic and Health Survey found an association with the type of cooking fuel used in a household and an increase in under-five mortality [30]. It is possible that exposure to indoor air pollution from cooking fuels could be a major contributing factor to our study`s under-five mortality.

It is also possible that the majority of RTIs in the adult population could potentially be undiagnosed active tuberculosis (TB) infections. Of the individuals who reported an RTI in the last 30 days, 86.8% reported that their RTIs lasted longer than 2 weeks and of these individuals, 65.4% reported at least one or more of the following symptoms: coughing up blood (15.4%), unexplained weight loss (23.1%), or night sweats (26.9%). While TB mortality rates have been declining in Myanmar [31], there is almost no data on TB for the Lahe Township region. It is possible that our study could highlight overlooked TB cases that are seldom reported.

Although the comparison was not significant in our study, there was a large point estimate difference in mortality between urban and rural females that could suggest more research is needed on female mortality in rural Lahe Township. Rural females were four times more likely to die than urban females. Unfortunately, while the difference in all-cause mortality point estimates between the two groups is large, we were unable to explore if there was a difference in causes of death between urban and rural females due to the limited sample size of causes of death for urban women. Our sex-specific results for mortality are slightly different than the sex-specific results for mortality listed in the Myanmar census [7]. Both our study and the Myanmar census demonstrate that males have a higher mortality rate compared to females overall. However, the Myanmar census only stratifies their results at the district level, not the township level, and not by the same urban/rural definition used in this study. Khamti district, which is the district that Lahe Township is part of, contains several urban areas with the biggest township having a population of over 250,000 individuals [5]. By comparison, Lahe Township has a population of approximately 50,000 individuals. It is possible that our mortality results are not directly comparable to the mortality results listed in the Myanmar census because our study investigates a rural population at the township level, while the census only reports the smallest geographic level of analysis at the district level. Although our results were not significant, the difference in the size of the point estimates between urban vs. rural females could suggest that it is possible a potentially neglected difference in the mortality of urban females compared to rural females has been overlooked because mortality data at the township level has not been rigorously analyzed yet.

### Maternal health

Our results suggest that Lahe Township could benefit from more health promotion activities that focus on family planning and contraception. Over 35.0% of women between the ages of 15 and 49 gave birth in the last two years, and 10.1% of women in the same age group were currently pregnant at the time of the survey. Less than half of women who were pregnant during the previous two years visited a health care provider for at least one antenatal care visit and only 2.2% of women who were pregnant during the previous two years received the recommended 4 antenatal care visits. This is considerably lower than Myanmar`s national average of 50.8% of rural mothers receiving at least 4 antenatal care visits cited by UNICEF in 2016 [32]. The most likely reasons why so many expectant mothers are not receiving antenatal care is probably due to a combination of lack of access to maternal and child health care and the difficulty in accessing transportation to a health facility which can provide antenatal services. Transportation is the biggest reason for not accessing health care cited in this survey and it is likely that in addition to impeding health care access, difficulty in securing transportation probably also negatively impacts receiving antenatal care [33][34].

The birth rate in Lahe Township was higher than expected. Assuming that Lahe Township`s population was relatively constant during the last 2 years and that half of the women who gave birth in the last 2 years did so within the previous year, our crude birth rate would be approximately 24.4 births per 1,000 individuals per year, which is higher than Myanmar`s overall national birth rate of 17.8 births per 1,000 individuals per year [8]. Additionally, 16.7% of the Lahe Township population is under the age of 5 and the median household size is 6. Possibly contributing to the high fertility in Lahe Township is the phenomenon that 89.0% of women who gave birth in the last 2 years reported not having a main source of contraception before they became pregnant. High fertility rates and large family sizes in rural areas is consistent with the fertility rates reported in the Myanmar census [8]. While the national average number of children per women for Myanmar is 2.5 children per woman of childbearing age, these numbers vary highly by socioeconomic factors and geographic region. The average number of children per woman for the three Nagaland Townships in the Sagaing region, Leshi, Nanyun, and Leshi, are 5.27, 4.32, and 6.14 respectively. By comparison, the number of children per women for Myanmar’s two most populated cites, Mandalay and Yangon, are 2.25 and 1.97 [5][8][35]. In addition to high birth and fertility rates, most women reported delivering their child at home (85.2%), with a significantly higher percentage of urban women delivering in a healthcare setting than rural women. Our study`s results suggest that women might not be able to access sufficient family planning and reproductive health care and that women could be unable to access a health clinic to give birth in.

### Access to health care

Less than half (46.7%) of households surveyed reported the ability to access a hospital if there was a need to. The inability to access a hospital varied greatly according to an individual’s urban / rural status. Over 80% of individuals in an urban area reported they were able to access a hospital if needed compared to only 42.8% of individuals in rural areas. The type of clinics an individual accessed when sick also varied according to urban / rural status. Almost all urban individuals (94.4%) preferred to visit Lahe`s Maternal and Child Health (MCH) center, whereas the types of clinics utilized in rural areas was more diverse, with the majority of individuals accessing either a rural health center / sub-center (46.1%), Lahe`s MCH center (22.4%), local midwife (6.8%), and the regional Myanmar military hospital (6.5%).

Much of the difference in the ability to access health care between urban and rural populations is because of either difficulties securing transportation or the cost of transportation. Transportation as a barrier to accessing health care has been a long-standing and persistent problem in rural Myanmar [36] [37]. Infrastructure for transportation is distributed unevenly with a lack of vehicles, limited number of roads, and dangerous terrain conditions common in rural areas. All of which make travel in the rural areas of Myanmar disproportionately expensive for the poor and vulnerable individuals living there [37]. In addition to transportation representing a significant barrier for individuals trying to access healthcare, the high cost of transportation is also a contributing factor for the low retention rate of health care staff posted to rural areas. This double effect of transportation as a barrier to accessing health care and a barrier to recruiting health care staff greatly increases the risk of individuals not being able to receive health care when they need it.

### Water and sanitation

Our results reflect critical water and sanitation issues in Lahe Township. Roughly 1 out of 8 households reported not having regular access to a source of water and more than 1 out of 4 households took over 5 minutes to access a source of water. Almost 20% of households did not treat their drinking water before consumption, which could represent a potential health hazard given that 73.2% of the population receives their water from a stream/lake/pond/river. Our findings suggest a possible future scenario where waterborne illnesses become more common. The type of latrine a household used was dependent on a household`s urban/rural status. The majority of urban individuals (94.4%) utilized an improved pit latrine, but only 30.3% of rural households utilized an improved pit latrine. While only 5.6% of urban households reported no latrine facilities, 23.3% of rural households reported no latrine facilities. Only 14.6% of households reported using soap and water after using a latrine and 39.6% of household reported not washing their hands at all after using the latrine. The lack of proper latrines combined with inadequate hand washing suggests that water and sanitation in Lahe Township could be why watery diarrhea was the second most commonly reported illness.

### Nutritional status

Our results suggest that there are very few cases of acute malnutrition in Lahe Township. The median MUAC score for children between the ages of 6 to 59 months was 145.0 (IQR: 138.0–153.0). Less than 2% of MUAC scores were classified as moderate acute malnutrition (1.5%) or severe acute malnutrition (0.3%) and there were no cases of oedem found through this survey. All cases of severe acute malnutrition and moderate acute malnutrition were found in rural households and no cases of were found in urban households. It is possible the reason so few cases of malnutrition were found in Lahe Township is because of increased involvement of the World Food Program in the Nagaland region after a nutritional assessment conducted in 2015 highlighted that seven percent of children were found to be malnourished and that the majority of children were suffering from intestinal parasitic infections [38][39]. While the World Food Program is planning on conducting a formal follow up survey to assess the effectiveness of their interventions in 2019, it is possible their nutritional programs have been effective and our survey data is reflecting this.

### Limitations

Although care was taken during the planning and implementation of this survey, there were a number of limitations that may affect the validity of the study. Unfortunately, because the causes of mortality are self-reported we were unable to determine the results of deaths classified as “other” beyond what was reported by the household. It is possible that our mortality rates are lower than expected due to households under reporting deaths in the family. Death is a highly hermetic event in Naga culture, and despite our survey staff being recruited locally, it is possible that households are uncomfortable talking about death to people outside their village. The unwillingness of the Naga community to discuss mortality could also partially explain why a large proportion of deaths were classified as “Other” or had no cause reported. The unwillingness to discuss mortality could also explain why we were unable to determine if maternal mortality was a factor associate with the differences in mortality between urban and rural females in Lahe Township. No cases of maternal death were reported, which was unexpected because according to United Nations Population Fund and the 2014 Myanmar census the maternal mortality rate for Myanmar is second highest in the Association of Southeast Asian Nations region (282 deaths per 100,000 births) [7][40].

It is possible that our morbidity results are under reported due to the head of the household not knowing the details of each household member’s health status. This might be particularly true for male household heads not being fully aware of maternal and child health issues of the female household members. Additionally, if the household head who was surveyed was not the main caregiver for the children living in the house, it is possible that they would not be fully aware of each child`s health status. To mitigate these limitations, each survey team consisted of one male and one female surveyor, in order for females to talk to the women of the household about issues regarding maternal and child health.

We were unable to establish a household`s duration of residence in Lahe Township, which could potentially affect our results if there was a high rate of mobility in and out of Lahe Township within the study`s recall period. However, the majority of households have resided in their village for several years, particularly if the household has children. Additionally, if a household was to move to another village, it is almost always within the township, and if a household moves out of the township, itis almost never outside of the Nagaland region. Therefore, while it is possible that the mobility of study participants could affect the results of this study, we believe that a participants movement between townships will have a minor effect on our results.

While we recognize the potential importance of seasonality in mortality, we were unable to determine the time of year of death for most mortality cases as most households did not report this. As such, we were unable to determine if the mortality rate was disproportionately affected by the time of year.

Questions on utilizing health care might not have been fully understood, particularly in rural areas. There is only one hospital and one Maternal and Child Health center in Lahe Township and it is located in Lahe town. We know that accessing these health centers is difficult since less than half of the households surveyed said they could access a hospital if they need to. Yet despite the difficulties in accessing Lahe town, almost a third of respondents reported visiting the Maternal and Child Health center in Lahe town when they were sick. It is possible that individuals are confusing accessing the Maternal and Child Health center with accessing a midwife, given that midwives are interspersed throughout the 6 rural health centers and 30 sub-centers of Lahe Township. The potential confusion over the nomenclature of the type of health care people can access could possible affect the results of our survey.

This study is missing information about household births during the last 2 years for 148 households (16.8% of houses). It is unknown if the missing information could have potentially influenced the results of pregnancy related questions during this study. It is highly likely that in reality the missing information represents households that have not experienced a pregnancy during the last two years and have chosen to skip this question because child birth is a celebrated event in Naga culture, however due to no answer being recorded for the question we can only assume that the data is missing.

## Conclusions

Our study suggests that mortality in Lahe Township is over the emergency threshold set by SPHERE standards and urgent attention is needed. Given that RTIs were the most commonly reported cause of death, more research is needed on RTIs in Myanmar`s Nagaland region. Improvements in transportation could significantly reduce the barriers blocking individuals from accessing healthcare and reproductive services. Water and sanitation should be improved on in order to increase public health infrastructure capacity.
